# Surgical approaches for achalasia and obesity: a systematic review and patient-level meta-analysis

**DOI:** 10.1007/s00423-023-03143-5

**Published:** 2023-10-16

**Authors:** Stephen Kunz, Hamza Ashraf, Christopher Klonis, Sarah K. Thompson, Ahmad Aly, David S. Liu

**Affiliations:** 1 Upper Gastrointestinal Surgery Unit, Division of Surgery, Anaesthesia, and Procedural Medicine, Austin Health, 145 Studley Road, Heidelberg, VIC 3084 Australia; 2 Department of Surgery, Austin Precinct, Austin Health, The University of Melbourne, 145 Studley Road, Heidelberg, Victoria 3084 Australia; 3 General and Gastrointestinal Surgery Research and Trials Group, Department of Surgery, Austin Precinct, Austin Health, The University of Melbourne, 145 Studley Road, Heidelberg, Victoria 3084 Australia; 4 Discipline of Surgery, College of Medicine and Public Health, Flinders University, Bedford Park, South Australia 5042 Australia; 5 Division of Cancer Surgery, Peter MacCallum Cancer Centre, 305 Grattan Street, Melbourne, Victoria 3000 Australia

**Keywords:** Obesity, Achalasia, Bariatric surgery, Eckardt score

## Abstract

**Purpose:**

Synchronous and metachronous presentations of achalasia and obesity are increasingly common. There is limited data to guide the combined or staged surgical approaches to these conditions.

**Methods:**

A systematic review (MEDLINE, Embase, and Web of Science) and patient-level meta-analysis of published cases were performed to examine the most effective surgical approach for patients with synchronous or metachronous presentations of achalasia and obesity.

**Results:**

Thirty-three studies with 93 patients were reviewed. Eighteen patients underwent concurrent achalasia and bariatric surgery, with the most common (*n* = 12, 72.2%) being laparoscopic Heller’s myotomy (LHM) and Roux-en-Y gastric bypass (RYGB). This combination achieved 68.9% excess weight loss and 100% remission of achalasia (mean follow-up: 3 years). Seven (6 RYGB, 1 biliopancreatic diversion) patients had bariatric surgery following achalasia surgery. Of these, all 6 RYGBs had satisfactory bariatric outcomes, with complete remission of their achalasia (mean follow-up: 1.8 years). Sixty-eight patients underwent myotomy following bariatric surgery; the majority (*n* = 55, 80.9%) were following RYGB. In this scenario, per-oral endoscopic myotomy (POEM) achieved higher treatment success than LHM (*n* = 33 of 35, 94.3% vs. *n* = 14 of 20, 70.0%, *p* = 0.021). Moreover, conversion to RYGB following a restrictive bariatric procedure during achalasia surgery was also associated with higher achalasia treatment success.

**Conclusion:**

In patients with concurrent achalasia and obesity, LHM and RYGB achieved good outcomes for both pathologies. For those with weight gain post-achalasia surgery, RYGB provided satisfactory weight loss, without adversely affecting achalasia symptoms. For those with achalasia after bariatric surgery, POEM and conversion to RYGB produced greater treatment success.

**Supplementary Information:**

The online version contains supplementary material available at 10.1007/s00423-023-03143-5.

## Introduction

While achalasia and obesity uncommonly co-occur [1], there is increasing epidemiological evidence linking these two conditions [2–4]. Firstly, achalasia is more prevalent in the surgery-naive obese population than in the general population [5–7]. Secondly, obesity may manifest following interventions for achalasia [8, 9]. Thirdly, bariatric surgery appears to be a risk factor for post-operative achalasia [10–13]. Finally, the rising incidence of obesity and associated bariatric procedures suggest that the phenomena of achalasia and obesity will become increasingly prevalent [14]. The complex interplay between these two pathologies and their associated treatment options highlights the need to consider both simultaneously when planning surgery for either.

The aims of surgical treatment for achalasia and obesity are to improve quality of life, alleviate symptoms, prevent long-term sequalae, promote healthy weight loss, and reduce obesity-related co-morbidities. Currently, there is a paucity of data to guide the optimal surgical approach to synchronous or metachronous presentations of achalasia and obesity. This is largely owing to the low prevalence of both conditions in clinical practice. Nonetheless, commentaries from Wesp et al. and Shenfine drew the conclusion that simultaneous procedures for both pathologies were feasible [3, 4]. Additionally, the management of achalasia following Roux-en-Y gastric bypass (RYGB) was explored by Aiolfi et al.’s, which supported either laparoscopic Heller’s myotomy (LHM) or per-oral endoscopic myotomy (POEM) [11].

Despite the above narratives, a systematic appraisal of available evidence for the management of achalasia and obesity has not been undertaken. Accordingly, we performed a systematic review and patient-level meta-analysis of published literature to help guide decision-making around combined (for the synchronous presentation of achalasia and obesity) or staged (for the metachronous presentation of achalasia after obesity surgery or obesity after achalasia surgery) surgical approaches to achalasia and obesity.

## Methods

### Study identification and screening process

We performed a systematic review and patient-level meta-analysis according to the Preferred Reporting Items for Systematic Reviews and Meta-Analyses (PRISMA) guidelines [15]. We conducted a comprehensive search of three databases (MEDLINE, EMBASE, and Web of Science) to identify eligible studies published between 1 January 2000 and 18 July 2022. We used search terms related to achalasia and obesity surgery (Supplementary Table S1). Our search was not language restricted. Furthermore, the bibliographies of each publication were manually reviewed for studies that were not captured in the initial search. References were imported into EndNote™ X8 (Clarivate, Philadelphia, PA, USA), and duplicates were removed. These were then manually screened by two authors (HA and CK) by title and abstract for inclusion. Studies which satisfied the initial screen underwent full-text review. Any uncertainties were resolved by a third author (DL).

### Eligibility criteria

Studies selected for final review included patients who underwent surgery (endoscopic, laparoscopic, or open) for obesity and achalasia performed either concurrently or in a staged approach. We included all publications (case reports, case series, and cohort studies) that reported on outcomes of weight loss or achalasia treatment.

### Outcomes and definitions

The Eckardt score is a 4-item scale measuring the extent of (1) weight loss in kg, (2) chest pain, (3) regurgitation, and (4) dysphagia for patients with achalasia. Each item is graded on a score of 0–3, with a maximum score of 12. Typically, scores > 3 are suggestive of active achalasia [1]. Accordingly, treatment success for achalasia was defined in this study as an Eckardt score ≤ 3, improvement in Eckardt score ≥ 1 when baseline score < 3 or symptomatic improvement with no re-intervention [1]. Treatment failure for achalasia was defined as those who did not meet the above criteria and had persisting or relapsed symptoms (dysphagia, regurgitation, and chest pain) at follow-up. Weight loss outcomes were defined as percentage excess weight loss (%EWL), percentage body mass index loss (%BMIL), or percentage total body weight loss (%TBWL).

### Individual patient data extraction

As all eligible publications were either case reports or case series describing individual cases, we were able to extract individual patient data with sufficient levels of data accuracy and completeness. Where there were missing data, as documented in Table 2 and Supplementary Tables S3 and S4, we attempted to contact the original investigators for clarification. However, due to a lack of response, these data points remained missing. Data was extracted independently by two authors (HA and CK) using a standardized electronic proforma. Patient-level meta-analysis was performed by two other authors (DL and SK). Study characteristics extracted included journal, author, year published, country of origin, study design, and number of patients. Patient characteristics extracted included age, gender, body mass index (BMI), achalasia subtype, previous obesity or achalasia treatment, time between achalasia and obesity surgeries, as well as admission data including length of stay, operation type, myotomy length, post-operative complications, follow-up duration, and treatment outcomes.

### Assessment of bias, methodological quality, and synthesis

We employed the proposed methods and tool by Murad et al. to assess for the presence of bias (Supplementary Table S2), methodological quality, and synthesis of available case reports and case series which contributed to this patient-level meta-analysis.

### Data analysis

Analyses were performed using Prism v9 (GraphPad Software, San Diego, CA, USA). Study and patient-level data were summarized using descriptive statistics. Patient-level meta-analysis was performed, comparing categorical variables using Fisher’s exact test, and continuous variables using Student’s t-test. Statistical significance was defined as a two-tailed *p* < 0.05 and 95% confidence interval (CI) around the odds ratio (OR) that did not cross one.

## Results

### Study selection and characteristics

**Fig. 1:**
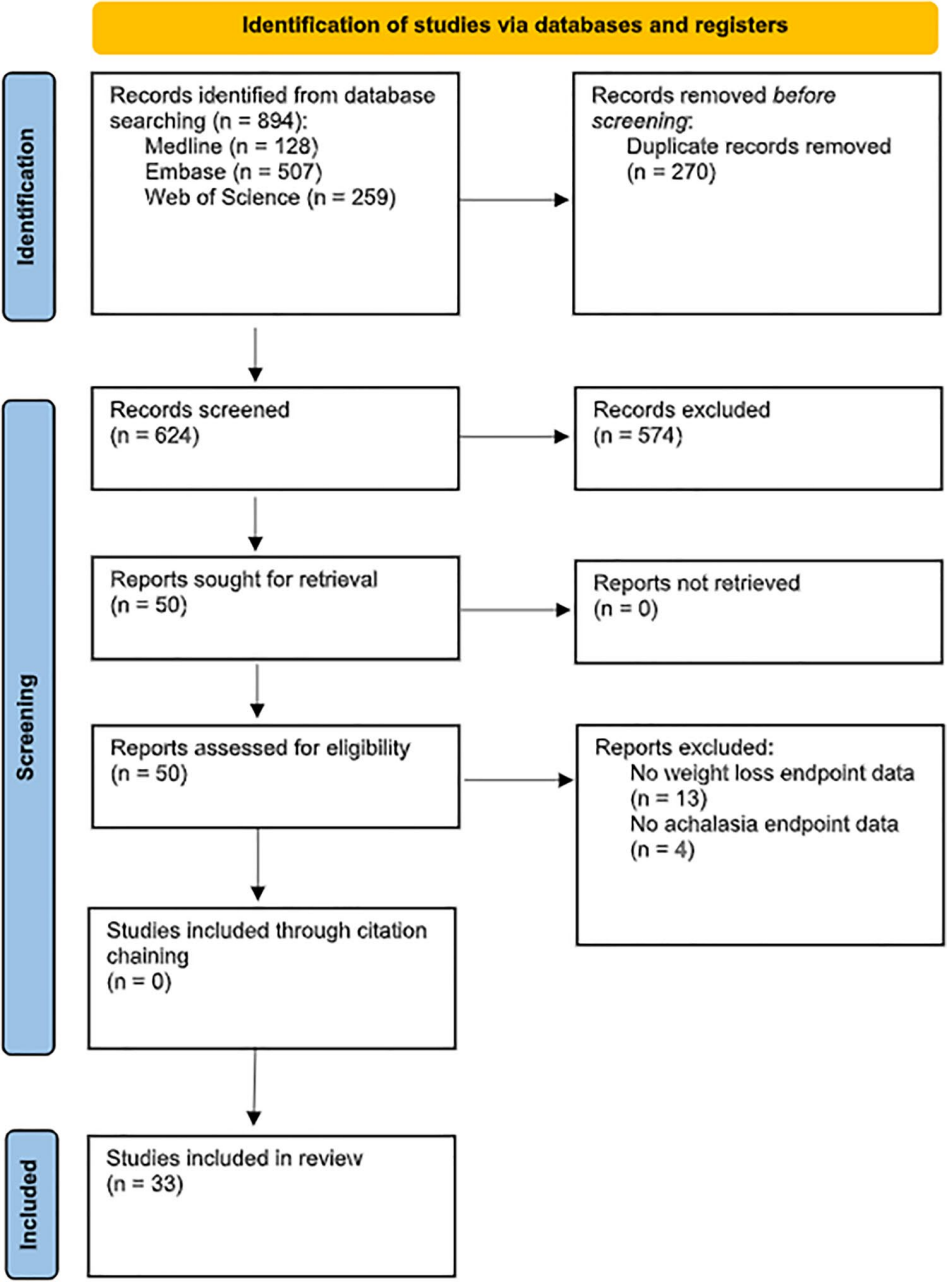
PRISM flowchart for systematic review and meta-analysis

Our systematic review identified a total of 894 studies (Fig. 1). Following screening, 33 were included in the final meta-analysis. All 33 publications were either case reports or case series that described in detail the history, investigations, treatments, and outcomes of 93 patients. As a result, a patient-level meta-analysis using descriptive and comparative statistics was performed. The assessment of potential bias, methodological quality, and synthesis for these case reports and case series are detailed in Supplementary Table S2. All included studies reached a high level of quality for case ascertainment, causality, and reporting. Some deficiencies were noted in case selection as it was unclear in some studies whether the reported case represented the whole experience of that center.

### Concurrent surgery for obesity and achalasia

In total, 18 patients were identified from 10 studies (Table 1 and Supplementary Table S3) [16–25], with a mean (SD) age of 47.9 (14.5) years. Of these, 12 were female, with a mean (SD) body mass index (BMI) of 45.9 (6.7) kg/m^2^. Four patients had prior pneumatic dilatation for their achalasia, one of whom also received a botox injection (Supplementary Table S3). Overall, thirteen (72.2%) patients underwent concurrent LHM and RYGB. At a mean (SD) follow-up of 27.5 (23.9) months, 12 patients (data was unavailable for 1 patient) achieved satisfactory weight loss, and all had remission of their achalasia symptoms (Table 1). There are potential pitfalls and technical considerations when combining LHM with RYGB [3, 4, 11]. A myotomy first approach allows any mucosal perforation to be easily repaired and covered with a fundoplication. The surgeon then has the option of deferring the RYGB to another day. Alternatively, if the RYGB has already been created, the gastric remnant may be used to cover the site of mucosal perforation. Secondly, during the creation of the gastric pouch for an RYGB, the passage of an endoscope under vision rather than blindly with a bougie may reduce the risk of inadvertent perforation of the myotomized esophagus. Thirdly, when fashioning the gastro-jejunostomy of an RYGB, care must be taken to avoid stapling across the myotomized stomach to minimize the risk of anastomotic leakage.

**Table 1:** Surgery for concurrent obesity and achalasia

Publication and patient details	Overall cohort (*N* = 18)
Case details	
Reports, *n* (%)	10
Publication year, *n* (%)	
≤ 2019	10 (55.6)
> 2019	8 (44.4)
Study origin, *n* (%)	
USA	7 (38.9)
Non-USA	11 (61.1)
Demography	
Age, years, mean (SD)	47.9 (14.5)
Gender, *n* (%)	
Female	12 (66.7)
Male	6 (33.3)
Body mass index, kg/m^2^, mean (SD)	45.9 (6.7)
Body mass index 40–50	13 (72.2)
Body mass index 51–60	5 (27.8)
Treatment details	
Achalasia subtype, *n* (%)	
1	4 (22.2)
2	8 (44.4)
3	2 (11.1)
Not documented	4 (22.2)
Previous achalasia treatment, yes, *n* (%)	3 (16.7)
Length of stay, days, mean (SD)	3.2 (0.8)
Post-op complications, yes, *n* (%)	0 (0.0)
Follow-up duration	
≥ 6 months, n (%)	14 (77.8)
Months, mean (SD)	24.2 (21.2)
Operation performed, n (%)	
LHM + RYGB	13 (72.2)
LHM + SG	1 (5.6)
Open DS + HM + Dor fundoplication	2 (11.1)
Open subtotal gastrectomy + HM	1 (5.6)
Open total gastrectomy	1 (5.6)
Outcomes	
LHM + RYGB	
Weight loss	
% EWL, mean (SD) | *n* | follow-up, mean months (SD)	68.9 (18.8) | 9 |35.1 (22.7)
% BMIL, mean | *n* | follow-up, months	25.5 | 1 | 6.0
% BMIL, mean | *n* | follow-up, months	13.8 | 1 | 3.0
Actual weight loss, kg | *n* | follow-up, months	18.1 | 1 | 4.0
Achalasia remission, yes, *n* (%)	13 (100.0)
LHM + SG	
Weight loss, kg | *n* | follow-up, months	5 | 1 | 1
Achalasia remission, yes, *n* (%)	1 (100.0)
Open DS + HM + Dor fundoplication	
Weight loss, kg | *n* | follow-up, months	NR
Achalasia remission, yes, *n* (%)	2 (100.0)
Open subtotal gastrectomy + HM	
Weight loss, kg | *n* | follow-up, months	20 | 1 | 24.0
Achalasia remission, yes, *n* (%)	1 (100.0)
Open total gastrectomy	
Weight loss, kg | *n* | follow-up, months	24 | 1 | 8.0
Achalasia remission, yes, *n* (%)	0 (0.0)

*%BMIL* % body mass index loss, *%EWL* % excess weight loss, *DS* duodenal switch, *HM* Heller’s myotomy, *LHM* laparoscopic Heller’s myotomy, *NR* not reported, *RYGB* Roux-en-Y gastric bypass, *SG* sleeve gastrectomy, *SD* standard deviation

Please refer to Supplementary Table S3 for individual study details

### Surgery for obesity following achalasia surgery

Seven patients were identified from 3 studies (Table 2) [26–28], with a mean (SD) age of 40.6 (6.2) years. All patients had a prior myotomy (4 LHM, 3 POEM), with a mean (SD) time from myotomy to bariatric surgery of 16.1 (20.2) months. Of these, 6 (85.7%) patients underwent RYGB for obesity. At a mean (SD) follow-up of 21.2 (12.1) months, their mean (SD) %TBWL was 30.9 (14.4) %, and all 6 patients remained in achalasia remission at follow-up.

**Table 2:** Surgery for obesity following achalasia surgery

Author, year, country	Age (year)	Sex	BMI (Kg/m^2^)	Achalasia type	Previous achalasia treatment	Time myotomy-bariatric surgery (months)	Bariatric operation performed	Length of stay (days)	Post-op complication	Follow-up (months)	Long-term bariatric outcomes	Long-term achalasia outcomes
Crafts et al., 2021, USA [28]	Mean 45	-	-	2	LHM + Dor	60	Open RYGB	-	Nil	36	25.6%TBWL	Clinical success
		-	-	-	LHM	1	RYGB	-	GJ strictures	12	51.4%TBWL	Clinical success
		-	-	3	LHM	7	RYGB	-	Roux limb stricture	8	18.0%TBWL	Clinical success
		-	-	3	POEM	4	RYGB	-	Nil	36	28.6%TBWL	Clinical success
Bashir et al., 2019, USA [27]	37	F	55.3	2	POEM	12	RYGB	2	Nil	15	-	Clinical success
	29	F	46.1	1	POEM	19	RYGB	2	Nil	20	-	Clinical success
Herbella et al., 2005, Brazil [26]	38	F	43.2	-	LHM	10	BPD, cardioplasty, antrectomy, vagotomy	-	Nil	12	7.2%BMIL	Clinical failure

*BMI* body mass index, *BPD* biliopancreatic diversion, *LHM* laparoscopic Heller’s myotomy, *POEM* per-oral endoscopic myotomy, *RYGB* Roux-en-Y gastric bypass, *%TBWL* % total body weight loss, *%BMIL* % body mass index loss

### Surgery for achalasia following bariatric surgery

Overall, 68 patients were identified from 23 studies (Table 3 and Supplementary Table S4) [11, 16, 27–47], with a mean (SD) age of 51.8 (9.3) years. Of these, 47 (69.1%) were female, with a mean (SD) BMI of 36.5 (11.1) kg/m^2^. All achalasia cases were presented after bariatric surgery and were diagnosed using high-resolution or conventional manometry. Overall, 29 (42.6%) patients received prior endoscopic interventions for their achalasia, as detailed in Supplementary Table S4. The mean (SD) time from bariatric to achalasia surgery was 7.2 (3.5) years. Fifty-five (80.9%) patients had a prior RYGB, and 13 (19.1%) had previously undergone a restrictive bariatric procedure (9 sleeve gastrectomies [SG], 2 vertical band gastroplasties [VBG], and 2 duodenal switches [DS]). Of those with a prior RYGB, 35 (63.6%) underwent POEM for definitive treatment of achalasia, while 20 (36.4%) received a Heller’s myotomy. Of those with an underlying restrictive gastric anatomy, all 13 patients received a myotomy; however, 3 patients also underwent conversion to RYGB.

**Table 3:** Surgery for achalasia following bariatric surgery

Publication and patient details	Overall cohort (*N* = 68)	Clinical success (*N* = 56)	Clinical failure (*N* = 12)	Clinical success vs. failure		
				OR	95% CI	*p*-value
Reports, *n*	23					
Publication year, *n* (%)						
≤ 2019	37 (54.4)	32 (57.1)	5 (41.7)	1.87	0.53–6.05	0.36
> 2019	31 (45.6)	24 (42.9)	7 (58.3)	Ref		
Patient origin, *n* (%)						
USA	62 (91.2)	50 (89.3)	12 (100.0)	-	-	0.58
Non-USA	6 (8.8)	6 (10.7)	0 (0.0)	Ref		
Demography						
Age, years, mean (SD)	51.8 (9.3)	51.5 (9.0)	52.7 (10.9)	-	-	0.69
Gender, *n* (%)						
Female	47 (69.1)	42 (75.0)	5 (41.7)	4.20	1.12–14.34	**0.037**
Male	14 (20.6)	10 (17.9)	4 (33.3)	0.43	0.11–1.52	0.25
Not documented	7 (10.3)	4 (7.1)	3 (25.0)	0.23	0.06–1.07	0.10
Body mass index, kg/m^2^, mean (SD)	36.5 (11.1)	35.8 (10.0)	40.7 (16.8)	-	-	0.52
Treatment details						
Previous achalasia treatment, yes, *n* (%)	29 (42.6)	24 (42.9)	5 (41.7)	1.05	0.30–3.42	1.00
Previous bariatric surgery, *n* (%)						
RYGB	55 (80.9)	47 (83.9)	8 (66.7)	2.61	0.74–11.27	0.22
SG	9 (13.2)	5 (8.9)	4 (33.3)	0.20	0.04–0.77	**0.045**
VBG	2 (2.9)	2 (3.6)	0 (0.0)	-	-	1.00
DS	2 (2.9)	2 (3.6)	0 (0.0)	-	-	1.00
Achalasia subtype, *n* (%)						
1	19 (27.9)	17 (30.4)	2 (16.7)	2.18	0.47–10.70	0.34
2	32 (47.1)	27 (48.2)	5 (41.7)	1.30	0.37–4.22	0.68
3	15 (22.1)	10 (17.9)	5 (41.7)	0.30	0.08–1.11	0.07
Not documented	2 (2.9)	2 (3.6)	0 (0.0)	-	-	1.00
Time bariatric surgery-myotomy, years, mean (SD)	7.2 (3.5)	7.2 (3.3)	7.5 (4.4)	-	-	0.78
Myotomy length, cm, mean (SD)	10.0 (1.6)	10.1 (1.6)	9.6 (1.4)	-	-	0.41
Length of stay, days, mean (SD)	1.7 (1.1)	1.7 (1.2)	1.5 (0.5)	-	-	0.61
Post-op complications, yes, n (%)	7 (10.3)	5 (8.9)	2 (16.7)	0.49	0.08–2.77	0.42
Follow-up duration						
> 6 months, *n* (%)	59 (86.8)	48 (85.7)	11 (91.7)	0.55	0.05–3.67	1.00
Months, mean (SD)	16.1 (10.6)	15.7 (10.7)	21.5 (11.8)	-	-	0.07
Achalasia operation performed, *n* (%)						
RYGB to POEM	35 (51.5)	33 (94.3)	2 (5.7)	7.07	1.42–36.30	**0.021**
RYGB to surgical myotomy	20 (29.4)	14 (70.0)	6 (30.0)	Ref		
SG/VBG/DS to any myotomy and RYGB	3 (4.4)	3 (100.0)	0 (0.0)	-	-	0.09
SG/VBG/DS to any myotomy	10 (14.7)	6 (60.0)	4 (40.0)	Ref		

Values in bold indicate *p* < 0.05

*CI* confidence interval, *DS* duodenal switch, *OR* odds ratio, *POEM* per-oral endoscopic myotomy, *RYGB* Roux-en-Y gastric bypass, *SG* sleeve gastrectomy, *VBG* vertical band gastroplasty, *SD* standard deviation

Please refer to Supplementary Table S4 for individual study details

We compared the characteristics of patients who achieved treatment success following achalasia surgery versus those with treatment failure (Table 3). These two groups were comparable in their year of publication, country of origin, follow-up duration, age, BMI, previous achalasia treatments, mix of achalasia subtypes, time from bariatric to achalasia surgery, myotomy length, length of stay, and post-operative complications following achalasia surgery. We found that female gender was associated with treatment success (OR 4.20, 95% CI 1.12–14.34, *p* = 0.037), and prior SG was associated with treatment failure (OR 5.10, 95% CI 1.30–24.37, *p* = 0.045). Interestingly, of those with a prior RYGB, patients who underwent POEM achieved a significantly higher rate of clinical success than those who received a Heller’s myotomy (94.3% versus 70.0%, OR 7.07, 95% CI 1.42–36.30, *p* = 0.021). Furthermore, we identified that conversion to RYGB after a previous restrictive bariatric procedure during achalasia surgery was associated with a trend (*p* = 0.09) toward higher achalasia treatment success (Table 3).

## Discussion

In this systematic review and patient-level meta-analysis, we described for the first time the global surgical experience of managing achalasia and obesity.

### Concurrent surgery for obesity and achalasia

We found that LHM and RYGB were the most common approaches for concurrent treatment of obesity and achalasia. This may be for several reasons. Firstly, RYGB minimizes post-myotomy gastroesophageal reflux [48]. This is achieved through the creation of a small gastric pouch, exclusion of acid-producing gastric mucosa, decreased intra-gastric pressure, improved gastric drainage, and prevention of bile reflux [3]. Secondly, both procedures utilize the same patient positioning and port placement and thus can be conveniently performed together [3]. Thirdly, given that achalasia predisposes to carcinomas of the esophagus [1], RYGB retains the native stomach for future conduit formation.

Other combinations of bariatric and achalasia surgeries have significant limitations. For example, LHM and single anastomosis gastric bypass predispose to gastroesophageal bile reflux [49]. LHM and SG are synergistically refluxogenic [48, 50]. Additionally, SG precludes the stomach from future reconstruction after an esophagectomy. LHM and gastric banding are inherently counter-productive, as banding will likely reverse the effect of the myotomy. Substituting LHM with POEM is possible, but unnecessarily adds complexity to any bariatric procedure.

### Surgery for obesity following achalasia surgery

We found that RYGB was the bariatric procedure of choice for most authors following achalasia surgery. This is likely explained by the advantages described above. In patients who have undergone a POEM procedure, RYGB should be relatively straightforward, given the paucity of adhesions. In contrast, in the author’s experience, we found that RYGB after Heller’s myotomy is technically more challenging due to hiatal adhesions and the need to release the pre-existing fundoplication. For a pre-existing anterior fundoplication, this dissection may predispose to esophageal injury (particularly at the myotomy site) as well as contribute to fundal ischemia (particularly if the short gastric vessels were previously divided). Strategies to mitigate these risks include meticulous dissection, early use of endoscopy to define the myotomy site, and fundal resection following wrap release. If a mucosal breach occurred, the gastric remnant can be used to patch the area in addition to suture repair. We found that posterior fundoplication is less problematic in this regard. Another potential option is to leave the prior myotomy and fundoplication in place and perform the gastric bypass below the fundoplication rather than risk injury to the prior myotomized area during the reversal of the fundoplication.

### Surgery for achalasia following bariatric surgery

Our analysis suggests that POEM is more efficacious than Heller’s myotomy after RYGB. This is a novel and interesting finding, particularly given that both cohorts had comparable myotomy lengths. We postulate that perhaps an endoscopic approach may achieve a “more complete” myotomy since it is traversing virgin tissues relative to a transabdominal approach. In addition, identification of anatomical landmarks using an endoscope may be easier than on laparoscopy in this setting, thus facilitating an adequate myotomy. In this regard, we feel that endoscopy is a valuable adjunct to Heller’s approach to confirm the adequacy of the myotomy.

Moreover, our analysis has demonstrated that prior SG is a risk factor for treatment failure following achalasia surgery. Additionally, we found that conversion to RYGB after a previous restrictive bariatric procedure may improve treatment outcomes for achalasia. These findings are supported by studies demonstrating that restrictive bariatric procedures, particularly SG, significantly increase the risk of post-operative dysphagia, regurgitation, and reflux [13, 51–53]. These adverse effects may be interpreted by patients as a failure of their achalasia treatment.

We acknowledge several limitations within our analysis. Firstly, our findings are based on case reports and case series. While this has enabled a detailed patient-level meta-analysis, we are limited by a small sample size and publication bias. Nonetheless, due to the relatively low incidence of obesity and achalasia, it would be impractical to conduct prospective studies in this area. Secondly, we are unable to comment on the role of other endoscopic interventions for achalasia (e.g., botox injection and balloon dilatation) in our treatment algorithm. However, it is recognized that, unlike surgery, botox injection and balloon dilatation do not provide durable achalasia remission [54–56]. Moreover, this systematic review was intended to focus on surgical options for both achalasia and obesity. In this regard, there is a bias toward higher numbers of RYGB relative to other weight loss procedures, making comparison between different surgical options challenging. Thirdly, our findings need to be interpreted within the follow-up period available to us from each study. Finally, due to limited data, we could not meta-analyze or comment upon preoperative investigations, perioperative complications, and metabolic outcomes related to these surgical approaches.

## Conclusion

**Fig. 2:**
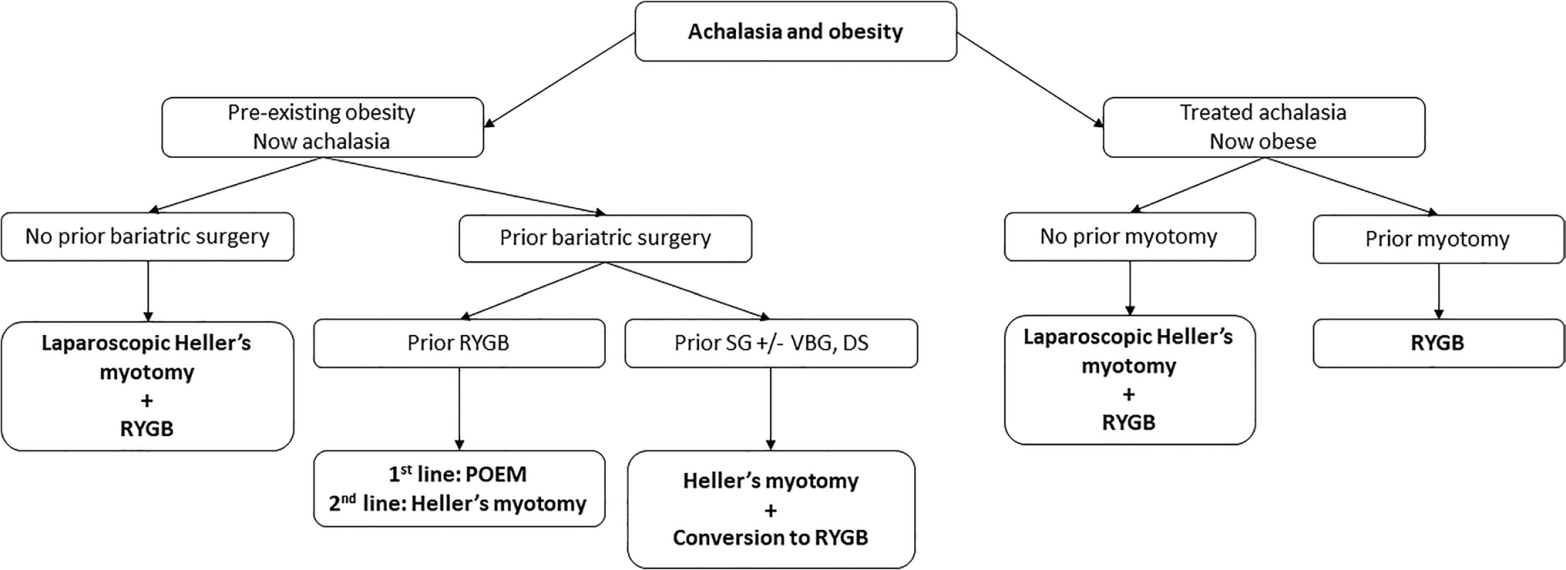
Proposed surgical treatment pathway for managing achalasia and obesity. DS: duodenal switch, POEM: per-oral endoscopic myotomy, SG: sleeve gastrectomy, RYGB: Roux-en-Y gastric bypass, VBG: vertical band gastroplasty

Our analysis suggests that a combination of LHM and RYGB adequately treats synchronous presentations of achalasia and obesity. For patients with obesity following achalasia surgery, RYGB enables weight loss without adversely affecting achalasia symptoms. For patients who develop achalasia after bariatric surgery, POEM and conversion to RYGB are associated with the highest rate of treatment success for achalasia. Within the context of available evidence and analysis, we propose a flow diagram to help guide decision-making around combined or staged operative approaches to synchronous or metachronous presentations of achalasia and obesity (Fig. 2).

### Supplementary Information

Below is the link to the electronic supplementary material.

Supplementary file1 (DOCX 128 KB)
