# Supplementary material for: Surgical approaches for achalasia and obesity: a systematic review and patient-level meta-analysis
Source: Langenbecks Arch Surg. 2023 Oct 16;408(1):403. doi: 10.1007/s00423-023-03143-5 (PMC10579133; doi:10.1007/s00423-023-03143-5)
Supplement: Supplementary file 1 — Supplementary file1 (DOCX 128 KB) [file 423_2023_3143_MOESM1_ESM.docx]

**Supplementary material**

**Table S1**. Systematic review search strategy

| **Search terms** | **Search strategy** |
| --- | --- |
| 1. Obesity 2. Morbid Obesity 3. Bariatrics 4. Bariatric surgery 5. Gastric bypass 6. Roux-en Y gastric bypass 7. Achalasia 8. Esophageal achalasia 9. Myotomy 10. Heller Myotomy 11. Peroral endoscopic myotomy 12. Esophagus function disorder 13. Cardioesophagomyotomy 14. Esophagus myotomy 15. Duodenal switch | **Theme 1: obesity**  - Obesity OR morbid obesity  - Bariatrics OR bariatric surgery  – Gastric bypass OR roux-en-y gastric bypass  - Gastroplasty  - Duodenal switch  **Theme 2: achalasia**  - Achalasia OR esophageal achalasia  - Myotomy OR Heller myotomy OR peroral endoscopic myotomy OR cardioesophagomyotomy OR esophagus myotomy  Items within each theme combined with ‘OR’, then both themes combined with ‘AND’ |

**Table S2.** Assessment of methodological quality and synthesis of case series and reports

| **Author/year** | **Q1** | **Q2** | **Q3** | **Q4** | **Q5** | **Q6** | **Q7** | **Q8** | **Assessment criteria**  **Selection**   1. Does the patient(s) represent(s) the whole experience of the investigator (centre)? No/Unclear=0 \| Yes=1   **Ascertainment**   1. Was the exposure adequately ascertained?   No=0 \| Yes=1   1. Was the outcome adequately ascertained?   No=0 \| Yes=1  **Causality**   1. Were other alternative causes that may explain the observation ruled out?   Yes=0 \| No=1   1. Was there a challenge, rechallenge phenomenon?   Not applicable=3   1. Was there a dose–response effect?   Not applicable=3   1. Was follow-up long enough for outcomes to occur?   No=0 \| Yes=1  **Reporting**   1. Is the case(s) described with sufficient details to allow practitioners to make inferences related to their own practice?   No=0 \| Yes=1 |
| --- | --- | --- | --- | --- | --- | --- | --- | --- | --- |
| *Studies describing surgery for obesity following achalasia surgery* | | | | | | | | |  |
| Crafts, 2021,[1] | 1 | 1 | 1 | 1 | 3 | 3 | 1 | 1 |  |
| Bashir, 2019,[2] | 1 | 1 | 1 | 1 | 3 | 3 | 1 | 1 |  |
| Herbella, 2005,[3] | 0 | 1 | 1 | 1 | 3 | 3 | 1 | 1 |  |
| *Studies describing concurrent surgery for obesity and achalasia* | | | | | | | | |  |
| Velotti, 2021,[4] | 0 | 1 | 1 | 1 | 3 | 3 | 1 | 1 |  |
| Ithurralde-Argerich, 2021,[5] | 1 | 1 | 1 | 1 | 3 | 3 | 1 | 1 |  |
| Friedman, 2017,[36] | 0 | 1 | 1 | 1 | 3 | 3 | 1 | 1 |  |
| Fisichella, 2015,[6] | 0 | 1 | 1 | 1 | 3 | 3 | 1 | 1 |  |
| Hagen, 2010,[7] | 0 | 1 | 1 | 1 | 3 | 3 | 0 | 1 |  |
| Pinto, 2010,[8] | 0 | 1 | 1 | 1 | 3 | 3 | 0 | 1 |  |
| Leon, 2010,[9] | 0 | 1 | 1 | 1 | 3 | 3 | 1 | 1 |  |
| O’Rourke, 2007,[10] | 0 | 1 | 1 | 1 | 3 | 3 | 1 | 1 |  |
| Kaufman, 2005,[11] | 0 | 1 | 1 | 1 | 3 | 3 | 1 | 1 |  |
| Almorgy, 2003,[12] | 1 | 1 | 1 | 1 | 3 | 3 | 1 | 1 |  |
| *Studies describing surgery for achalasia following bariatric surgery* | | | | | | | | |  |
| Donatelli, 2021,[13] | 0 | 1 | 1 | 1 | 3 | 3 | 0 | 1 |  |
| Bomman, 2021,[14] | 1 | 1 | 1 | 1 | 3 | 3 | 1 | 1 |  |
| Kolb, 2020,[15] | 1 | 1 | 1 | 1 | 3 | 3 | 1 | 1 |  |
| Aiolfi, 2021,[16] | 0 | 1 | 1 | 1 | 3 | 3 | 1 | 1 |  |
| Crafts, 2021,[1] | 1 | 1 | 1 | 1 | 3 | 3 | 1 | 1 |  |
| Bashir, 2019,[2] | 1 | 1 | 1 | 1 | 3 | 3 | 1 | 1 |  |
| Kim, 2019,[17] | 0 | 1 | 1 | 1 | 3 | 3 | 1 | 1 |  |
| Casas, 2019,[18] | 0 | 1 | 1 | 1 | 3 | 3 | 1 | 1 |  |
| Aiolfi, 2019,[19] | 0 | 1 | 1 | 1 | 3 | 3 | 1 | 1 |  |
| Sanaei, 2019,[20] | 1 | 1 | 1 | 1 | 3 | 3 | 1 | 1 |  |
| Birriel, 2017,[21] | 0 | 1 | 1 | 1 | 3 | 3 | 0 | 1 |  |
| Luo, 2017,[22] | 0 | 1 | 1 | 1 | 3 | 3 | 1 | 1 |  |
| Masrur, 2016,[23] | 0 | 1 | 1 | 1 | 3 | 3 | 1 | 1 |  |
| Nguyen, 2016,[24] | 0 | 1 | 1 | 1 | 3 | 3 | 1 | 1 |  |
| Johnson, 2016,[25] | 0 | 1 | 1 | 1 | 3 | 3 | 1 | 1 |  |
| Boules, 2016,[26] | 1 | 1 | 1 | 1 | 3 | 3 | 1 | 1 |  |
| Torghabeh, 2015,[27] | 1 | 1 | 1 | 1 | 3 | 3 | 1 | 1 |  |
| Yang, 2014,[28] | 0 | 1 | 1 | 1 | 3 | 3 | 0 | 1 |  |
| Oh, 2014,[29] | 0 | 1 | 1 | 1 | 3 | 3 | 1 | 1 |  |
| Benavente-Chenhalls, 2011,[30] | 0 | 1 | 1 | 1 | 3 | 3 | 0 | 1 |  |
| Ramos, 2009,[31] | 0 | 1 | 1 | 1 | 3 | 3 | 1 | 1 |  |
| Cho, 2006,[32] | 0 | 1 | 1 | 1 | 3 | 3 | 1 | 1 |  |
| Almogy, 2003,[12] | 0 | 1 | 1 | 1 | 3 | 3 | 1 | 1 |  |

Assessment of methodological quality and synthesis of case series and reports was according to tools proposed by Murad et al. [33]

**Table S3.** Studies describing concurrent surgery for obesity and achalasia

| **Author,**  **year** | **Age**  **(Yr)** | **Sex** | **BMI** | **Achalasia**  **type** | **Previous**  **achalasia**  **treatment** | **Operation**  **performed** | **Myotomy**  **length**  **(cm)** | **Surgery**  **Time**  **(mins)** | **LOS** | **Postop**  **Complication,**  **Describe** | **Follow-up**  **(months)** | **Long term**  **weight**  **outcomes** | **Long term**  **achalasia**  **Outcomes** |
| --- | --- | --- | --- | --- | --- | --- | --- | --- | --- | --- | --- | --- | --- |
| Velotti et al  2021^[34]^ | 46 | F | 47.0 | 2 | PD x2 | RYGB+LHM | - | 115 | 5 | Nil | 6.0 | 25.5 %BMIL | Clinical success |
| Ithurralde-Argerich et al  2021^[35]^ | 52 | F | 50.0 | 2 | Nil | RYGB+LHM | - | - | 3 | Nil | 73.2 | 70.1 %EWL | Clinical success |
|  | 54 | F | 40.0 | 2 | Nil | RYGB+LHM | - | - | 3 | Nil | 63.8 | 74.4 %EWL | Clinical success |
|  | 31 | M | 43.0 | 2 | Nil | RYGB+LHM | - | - | 3 | Nil | 41.0 | 70.5 %EWL | Clinical success |
|  | 42 | M | 40.0 | 2 | Nil | RYGB+LHM | - | - | 3 | Nil | 41.2 | 98.1 %EWL | Clinical success |
|  | 56 | M | 40.0 | 2 | Nil | RYGB+LHM | - | - | 3 | Nil | 37.1 | 75.0 %EWL | Clinical success |
|  | 54 | F | 43.0 | 3 | Nil | RYGB+LHM | - | - | 3 | Nil | 24.5 | 81.3 %EWL | Clinical success |
|  | 23 | M | 43.0 | 3 | Nil | RYGB+LHM | - | - | 3 | Nil | 17.9 | 70.3 %EWL | Clinical success |
| Friedman et al  2017^[36]^ | 30 | F | 42.0 | 1 | Nil | RYGB+LHM | - | - | - | Nil | 4.0 | 18.1 kg WL | Clinical success |
| Fisichella et al  2015^[37]^ | 45 | F | 45.0 | 2 | Nil | RYGB+LHM | 8.5 | - | - | Nil | - | - | Clinical success |
| Hagen et al  2010^[38]^ | 69 | F | 40.0 | 1 | Nil | LSG+LHM | - | - | 4 | Nil | 1.0 | 5 kg WL | Clinical success |
| Pinto et al  2010^[39]^ | 49 | F | 51.1 | 2 | Nil | RYGB+LHM | 10.0 | - | 2 | Nil | 3.0 | 13.8 %BMIL | Clinical success |
| Leon et al  2010^[40]^ | 71 | M | 36.0 | - | PD x2 | Open TG | Nil | - | - | Nil | 8.0 | 24 kg WL | Clinical failure |
|  | 66 | M | 44.0 | - | Nil | Open STG+HM | - | - | - | Nil | 24.0 | 20 kg WL | Clinical success |
| O’Rourke et al  2007^[41]^ | 60 | F | 52.0 | - | PD, Botox | RYGB+LHM | 8.0 | - | 3 | Nil | 6.0 | 33.0 %EWL | Clinical success |
| Kaufman et al  2005^[42]^ | 25 | F | 58.0 | - | PD | RYGB+LHM | 7.0 | - | - | Nil | 12.0 | 47.6 %EWL | Clinical success |
| Almorgy et al  2003^[43]^ | 54 | F | 60.0 | 1 | Nil | Open DS+HM+Dor | - | - | - | Nil | 24 | - | Clinical success |
|  | 36 | F | 52.7 | 1 | Nil | Open DS+HM+Dor | - | - | - | Nil | 24 | - | Clinical success |

BMI: Body mass index, % BMIL: % BMI loss, %EWL: % Excessive weight loss, %TBWL: % Total body weight loss, DS+HM: Duodenal switch + Heller’s myotomy, LOS: length-of-stay, PD: pneumatic dilatation, STG+HM: Subtotal gastrectomy + Hellers’ myotomy, RYGB+LHM: Roux en-Y Gastric bypass + Laparoscopic Heller’s myotomy, TG: Total gastrectomy, (-): data not available

Clinical success: Eckardt ≤3 or improvement in Eckardt score ≥1 when baseline score <3, or symptomatic improvement with no need for re-intervention

**Table S4.** Studies describing the outcomes of achalasia surgery after bariatric surgery

| **Author,**  **Year, Country** | **Age**  **(Yr)** | **Sex** | **BMI** | **Achalasia**  **type** | **Previous**  **bariatric**  **surgery** | **Previous**  **achalasia**  **treatment** | **Time**  **bariatric – myotomy**  **(years)** | **Operation**  **performed** | **Myotomy length**  **(cm)** | **Surgery**  **Time**  **(mins)** | **LOS**  **(days)** | **Postop**  **Complication,**  **Describe** | **Follow**  **up**  **(months)** | **Long term**  **Achalasia**  **Outcomes** |
| --- | --- | --- | --- | --- | --- | --- | --- | --- | --- | --- | --- | --- | --- | --- |
| Donatelli  2021, France ^[44]^ | 58 | F | 22.0 | 1 | Lap band  SG, RYGB | Nil | 12.0 | POEM | 10 | - | 1 | Nil | 2.0 | Eckardt 9🡪1  Clinical success |
| Bomman  2021, USA^[45]^ | Mean 52.3 | F (12)  M (4) | Mean 33.6 | 1 (2)  2 (9)  3 (5) | RYGB (14)  SG (2) | Botox (5)  PD (5) | Mean 7.5  SD 4.2 | POEM (16) | Mean 10.2  SD 2.7 | - | Mean 1.4  SD 0.7 | Leak (1) | 12 | Clinical success (14) |
| Kolb  2020, USA^[15]^ | 54 | M | - | 1 | SG | LHM | 3.9 | POEM | 9.3 (Range 9-10) | 79.8  (Range  47-105) | 1 | Grade A GORD | 37.0 | Eckardt 9🡪3  Clinical success |
|  | 68 | M | - | 2 | SG | Botox | 5.9 | POEM |  |  | 1 | Candida esophagitis | 21.0 | Eckardt 6🡪6  Clinical failure |
|  | 53 | F | - | 2 | SG | Botox, LHM | 13.8 | POEM |  |  | 1 | Grade B GORD | 13.0 | Eckardt 7🡪2  Clinical success |
|  | 43 | F | - | 2 | RYGB | Nil | 8.0 | POEM |  |  | 1 | Nil | 21.0 | Eckardt 10🡪10  Clinical failure |
|  | 31 | M | - | 2 | RYGB | Botox | 0.6 | POEM |  |  | 1 | Nil | 33.0 | Eckardt 4🡪2  Clinical success |
|  | 37 | F | - | 2 | RYGB | Nil | 10.2 | POEM |  |  | 1 | Nil | 1.5 | Eckardt 10🡪2  Clinical success |
| Aiolfi  2021, Italy^[16]^ | 46 | F | 20.0 | 2 | SG | Nil | 4.0 | LHM+Dor | 8 | 85 | 2 | Nil | 12.0 | Eckardt 7🡪0  Clinical success |
| Crafts  2021, USA^[1]^ | Mean  60 .0 | - | - | 1 | RYGB | Stent, PD | 13.0 | POEM | - | - | - | GJ stricture | 12.0 | Clinical success |
|  |  | - | - | 2 | RYGB | Nil | 12.0 | LHM | - | - | - | Nil | 24.0 | Clinical failure |
|  |  | - | - | 3 | RYGB | Nil | 10.0 | LHM, HHR | - | - | - | Nil | 24.0 | Clinical failure |
|  |  | - | - | 1 | RYGB | Nil | 0.3 | LHM | - | - | - | Nil | 2.0 | Clinical success |
|  |  | - | - | 2 | SG | Nil | 1.0 | LHM | - | - | - | Nil | 5.0 | Clinical failure |
|  |  | - | - | 3 | SG | Nil | 6.0 | LHM, RYGB | - | - | - | GJ stricture | 24.0 | Clinical success |
|  |  | - | - | 2 | DS | Nil | 9.0 | LHM, HHR | - | - | - | Malnutrition | 36.0 | Clinical success |
| Bashir  2019, USA^[2]^ | 28 | F | 39.1 | 2 | RYGB | Nil | 7.0 | POEM | 12 (Range 10-13) | - | 2 | Nil | 4.0 | Eckardt 9🡪0  Clinical success |
|  | 32 | F | 28.5 | 2 | RYGB | Nil | 6.0 | POEM |  | - | 2 | Nil | 12.0 | Eckardt 9🡪0  Clinical success |
|  | 66 | F | 23.8 | 3 | RYGB | Nil | 18.0 | POEM |  | - | 2 | Nil | 12.0 | Eckardt 6🡪7  Clinical failure |
|  | 56 | F | 42.4 | 3 | RYGB | Nil | 2.0 | POEM |  | - | 2 | Nil | 6.0 | Eckardt 9🡪0  Clinical success |

**Table S4 continued**

| **Author,**  **Year, Country** | **Age**  **(Yr)** | **Sex** | **BMI** | **Achalasia**  **type** | **Previous**  **bariatric**  **surgery** | **Previous**  **achalasia**  **treatment** | **Time**  **bariatric – myotomy**  **(years)** | **Operation**  **performed** | **Myotomy length**  **(cm)** | **Surgery**  **Time**  **(mins)** | **LOS**  **(days)** | **Postop**  **Complication,**  **Describe** | **Follow**  **up**  **(months)** | **Long term**  **Achalasia**  **Outcomes** |
| --- | --- | --- | --- | --- | --- | --- | --- | --- | --- | --- | --- | --- | --- | --- |
| Kim  2019, USA^[17]^ | 47 | F | - | 3 | RYGB | LHM, PD | 3.0 | LHM | 8 | - | - | Nil | 12.0 | Clinical failure |
| Casas  2019, Brazil^[18]^ | 40 | F | 31.0 | 2 | RYGB | Nil | 1.0 | LHM+Dor | 7 | - | - | Nil | 6.0 | Clinical success |
| Aiolfi  2019, Italy^[46]^ | 51 | F | 25.7 | 2 | RYGB | PD | 9.0 | LHM | - | - | - | Nil | 24.0 | Eckardt 9🡪2  Clinical success |
| Sanaei  2019, USA, European^[20]^ | Mean 52.5 | F (8)  M (2) | - | 1 (5)  2 (4)  3 (1) | RYGB | Botox, PD (1)  PD (2)  LHM (1) | Median 7.5 | POEM (10) | Mean 11  SD 1.7 | Mean 72  SD 22.9 | Mean 1.5  SD 0.7 | Nil | 19.0 | Clinical success (10) |
| Birriel  2017, USA^[21]^ | 46 | F | 34.3 | 1 | RYGB | Nil | 4.0 | LHM+Dor | 8 | - | 2 | Nil | 1.0 | Clinical success |
| Luo  2017, USA^[22]^ | 67 | F | 24.9 | 3 | RYGB | Nil | 12.0 | POEM | 10 | - | 2 | Nil | 6.0 | Clinical success |
| Masrur  2016, USA^[23]^ | 52 | F | 39.9 | - | RYGB | Botox, PD | 14.0 | LHM | 6 | 78 | 2 | Nil | 5.0 | Clinical success |
| Nguyen  2016, USA^[24]^ | 66 | M | 36.2 | 3 | RYGB | Botox, PD | 3.0 | LHM | 12 | - | 3 | Nil | 12.0 | Clinical success |
| Johnson  2016, USA^[25]^ | 47 | F | 26.4 | 2 | RYGB | Nil | 8.0 | OHM+Dor | - | - | 1 | Nil | 8.0 | Clinical success |
| Boules  2016, USA^[26]^ | 63 | F | 37.7 | 2 | RYGB | Nil | Median  6.0 | LHM | - | - | - | - | 36.0 | Clinical success |
|  | 56 | M | 61.3 | 1 | RYGB | Nil |  | LHM | - | - | - | - | 36.0 | Clinical success |
|  | 52 | F | 63.4 | 1 | RYGB | Nil |  | OHM | - | - | - | - | 36.0 | Clinical success |
|  | 29 | F | 68.0 | 2 | RYGB | Botox, PD |  | LHM | - | - | - | - | 36.0 | Clinical failure |
|  | 47 | M | 54.0 | 1 | RYGB | Botox, PD |  | OHM | - | - | - | - | 36.0 | Clinical failure |
|  | 33 | M | 46.0 | 1 | RYGB | Nil |  | OHM | - | - | - | - | 36.0 | Clinical success |
|  | 43 | F | 64.6 | 2 | RYGB | PD |  | OHM | - | - | - | - | 36.0 | Clinical success |
|  | 56 | F | - | - | RYGB | PD |  | LHM | - | - | - | - | 36.0 | Clinical success |
| Torghabeh  2015, USA^[27]^ | 48 | F | 29.7 | 1 | RYGB | PD | 5.0 | LHM | 8 | - | 2 | Nil  GORD | 4.0  43.0 | Clinical success  Clinical failure |
| Yang  2014, USA^[28]^ | 64 | F | - | 2 | RYGB | Nil | - | POEM | 14 | - | 2 | Nil | 1.1 | Clinical success |
| Oh 2014, Singapore^[29]^ | 39 | F | 38.3 | 2 | SG | Nil | 3.0 | LHM, RYGB | 10 | - | - | Nil | 6.0 | Clinical success |

**Table S4 continued**

| **Author,**  **Year, Country** | **Age**  **(Yr)** | **Sex** | **BMI** | **Achalasia**  **type** | **Previous**  **bariatric**  **surgery** | **Previous**  **achalasia**  **treatment** | **Time**  **bariatric – myotomy**  **(years)** | **Operation**  **performed** | **Myotomy length**  **(cm)** | **Surgery**  **Time**  **(mins)** | **LOS**  **(days)** | **Postop**  **Complication,**  **Describe** | **Follow**  **up**  **(months)** | **Long term**  **Achalasia**  **Outcomes** |
| --- | --- | --- | --- | --- | --- | --- | --- | --- | --- | --- | --- | --- | --- | --- |
| Benavente-Chenhalls  2011, USA^[30]^ | 40 | F | 26.0 | 3 | VBG | Nil | 17.0 | LHM, RYGB | 7 | - | 2 | Nil | 1.0 | Clinical success |
| Ramos  2009, Brazil^[31]^ | 44 | F | 28.0 | 3 | RYGB | Nil | 4.4 | LHM | 6 | - | 2 | Nil | 6.0 | Clinical success |
| Cho  2006, USA^[32]^ | 78 | M | 36.1 | 1 | VBG | Botox, PD | 13.0 | LHM+Dor | - | - | 9 | Nil | 10.0 | Clinical success |
| Almogy  2003, USA^[43]^ | 57 | F | 43.3 | 1 | DS | Nil | 3.0 | OHM+Dor | - | - | - | Nil | 24.0 | Clinical success |

BMI: Body mass index, DS: Duodenal switch, LOS: length-of-stay, LHM: Laparoscopic Heller’s myotomy, OHM: Open Heller’s myotomy, PD: pneumatic dilatation, POEM: Per-oral endoscopic myotomy, SD: Standard deviation, SG: Sleeve gastrectomy, VBG: Vertical band gastroplasty. (-): data not available

Clinical success: Eckardt ≤3 or improvement in Eckardt score ≥1 when baseline score <3, or symptomatic improvement with no need for re-intervention

| **Section and Topic** | **Item #** | **Checklist item** | **Location where item is reported** |
| --- | --- | --- | --- |
| **TITLE** | | |  |
| Title | 1 | Identify the report as a systematic review. | Title |
| **ABSTRACT** | | |  |
| Abstract | 2 | See the PRISMA 2020 for Abstracts checklist. | Page 2 |
| **INTRODUCTION** | | |  |
| Rationale | 3 | Describe the rationale for the review in the context of existing knowledge. | Page 3 |
| Objectives | 4 | Provide an explicit statement of the objective(s) or question(s) the review addresses. | Page 3 |
| **METHODS** | | |  |
| Eligibility criteria | 5 | Specify the inclusion and exclusion criteria for the review and how studies were grouped for the syntheses. | Page 3-4 |
| Information sources | 6 | Specify all databases, registers, websites, organisations, reference lists and other sources searched or consulted to identify studies. Specify the date when each source was last searched or consulted. | Page 3-4 |
| Search strategy | 7 | Present the full search strategies for all databases, registers and websites, including any filters and limits used. | Page 3, Table S1 |
| Selection process | 8 | Specify the methods used to decide whether a study met the inclusion criteria of the review, including how many reviewers screened each record and each report retrieved, whether they worked independently, and if applicable, details of automation tools used in the process. | Page 3-4 |
| Data collection process | 9 | Specify the methods used to collect data from reports, including how many reviewers collected data from each report, whether they worked independently, any processes for obtaining or confirming data from study investigators, and if applicable, details of automation tools used in the process. | Page 4 |
| Data items | 10a | List and define all outcomes for which data were sought. Specify whether all results that were compatible with each outcome domain in each study were sought (e.g. for all measures, time points, analyses), and if not, the methods used to decide which results to collect. | Page 4 |
|  | 10b | List and define all other variables for which data were sought (e.g. participant and intervention characteristics, funding sources). Describe any assumptions made about any missing or unclear information. | Page 4 |
| Study risk of bias assessment | 11 | Specify the methods used to assess risk of bias in the included studies, including details of the tool(s) used, how many reviewers assessed each study and whether they worked independently, and if applicable, details of automation tools used in the process. | NA |
| Effect measures | 12 | Specify for each outcome the effect measure(s) (e.g. risk ratio, mean difference) used in the synthesis or presentation of results. | Page 4 |
| Synthesis methods | 13a | Describe the processes used to decide which studies were eligible for each synthesis (e.g. tabulating the study intervention characteristics and comparing against the planned groups for each synthesis (item #5)). | Page 4 |
|  | 13b | Describe any methods required to prepare the data for presentation or synthesis, such as handling of missing summary statistics, or data conversions. | Page 4 |
|  | 13c | Describe any methods used to tabulate or visually display results of individual studies and syntheses. | Page 4 |
|  | 13d | Describe any methods used to synthesize results and provide a rationale for the choice(s). If meta-analysis was performed, describe the model(s), method(s) to identify the presence and extent of statistical heterogeneity, and software package(s) used. | Page 4 |
|  | 13e | Describe any methods used to explore possible causes of heterogeneity among study results (e.g. subgroup analysis, meta-regression). | Page 4 |
|  | 13f | Describe any sensitivity analyses conducted to assess robustness of the synthesized results. | NA |
| Reporting bias assessment | 14 | Describe any methods used to assess risk of bias due to missing results in a synthesis (arising from reporting biases). | NA |
| Certainty assessment | 15 | Describe any methods used to assess certainty (or confidence) in the body of evidence for an outcome. | NA |
| **RESULTS** | | |  |
| Study selection | 16a | Describe the results of the search and selection process, from the number of records identified in the search to the number of studies included in the review, ideally using a flow diagram. | Page 5, Figure 1 |
|  | 16b | Cite studies that might appear to meet the inclusion criteria, but which were excluded, and explain why they were excluded. | Figure 1 |
| Study characteristics | 17 | Cite each included study and present its characteristics. | Page 5-6  Tables 1-3  Tables S2-3 |
| Risk of bias in studies | 18 | Present assessments of risk of bias for each included study. | NA |
| Results of individual studies | 19 | For all outcomes, present, for each study: (a) summary statistics for each group (where appropriate) and (b) an effect estimate and its precision (e.g. confidence/credible interval), ideally using structured tables or plots. | Pages 5-6  Tables 1-3 |
| Results of syntheses | 20a | For each synthesis, briefly summarise the characteristics and risk of bias among contributing studies. | Tables 1-3  Tables S2-3 |
|  | 20b | Present results of all statistical syntheses conducted. If meta-analysis was done, present for each the summary estimate and its precision (e.g. confidence/credible interval) and measures of statistical heterogeneity. If comparing groups, describe the direction of the effect. | Pages 5-6 |
|  | 20c | Present results of all investigations of possible causes of heterogeneity among study results. | NA |
|  | 20d | Present results of all sensitivity analyses conducted to assess the robustness of the synthesized results. | NA |
| Reporting biases | 21 | Present assessments of risk of bias due to missing results (arising from reporting biases) for each synthesis assessed. | NA |
| Certainty of evidence | 22 | Present assessments of certainty (or confidence) in the body of evidence for each outcome assessed. | Pages 5-6 |
| **DISCUSSION** | | |  |
| Discussion | 23a | Provide a general interpretation of the results in the context of other evidence. | Page 6-8 |
|  | 23b | Discuss any limitations of the evidence included in the review. | Page 6-8 |
|  | 23c | Discuss any limitations of the review processes used. | Page 6-8 |
|  | 23d | Discuss implications of the results for practice, policy, and future research. | Page 6-8 |
| **OTHER INFORMATION** | | |  |
| Registration and protocol | 24a | Provide registration information for the review, including register name and registration number, or state that the review was not registered. | Review not registered |
|  | 24b | Indicate where the review protocol can be accessed, or state that a protocol was not prepared. | NA |
|  | 24c | Describe and explain any amendments to information provided at registration or in the protocol. | NA |
| Support | 25 | Describe sources of financial or non-financial support for the review, and the role of the funders or sponsors in the review. | Title Page |
| Competing interests | 26 | Declare any competing interests of review authors. | Title Page |
| Availability of data, code and other materials | 27 | Report which of the following are publicly available and where they can be found: template data collection forms; data extracted from included studies; data used for all analyses; analytic code; any other materials used in the review. | NA |

**References for supplementary tables**

1. Crafts TD, Lyo V, Rajdev P, Wood SG. Treatment of achalasia in the bariatric surgery population: a systematic review and single-institution experience. Surgical endoscopy. 2021 Sep;35(9):5203-16.

2. Bashir U, El Abiad R, Gerke H, Keech J, Parekh K, Nau P. Peroral Endoscopic Myotomy Is Feasible and Safe in a Gastric Bypass Population. Obesity surgery. 2019 Nov;29(11):3523-26.

3. Herbella FA, Matone J, Lourenço LG, Del Grande JC. Obesity and symptomatic achalasia. Obesity surgery. 2005 May;15(5):713-5.

4. Velotti N, Vitiello A, Berardi G, Musella M. Roux-en-Y Gastric Bypass and Heller Myotomy: One-Step Surgical Treatment of Symptomatic Achalasia in a Morbid Obese Patient. Obesity surgery. 2021 Jul;31(7):3379-81.

5. Ithurralde-Argerich J, Rosner L, Faerberg A, Puma R, Ferro D, Cuenca-Abente F. Laparoscopic Heller Myotomy and Roux-en-Y Gastric Bypass as Treatment for Patients with Achalasia and Morbid Obesity: Outcomes in a Short Series of Patients. Journal of laparoendoscopic & advanced surgical techniques Part A. 2021 Jan;31(1):29-35.

6. Fisichella PM, Orthopoulos G, Holmstrom A, Patti MG. The surgical management of achalasia in the morbid obese patient. Journal of gastrointestinal surgery : official journal of the Society for Surgery of the Alimentary Tract. 2015 Jun;19(6):1139-43.

7. Hagen ME, Sedrak M, Wagner OJ, Jacobsen G, Talamini M, Horgan S. Morbid obesity with achalasia: a surgical challenge. Obesity surgery. 2010 Oct;20(10):1456-8.

8. Pinto G, Pestana J, Marin VD, Sendrea JG, Obregon F. Achalasia And Morbid Obesity: Simultaneous Management By Heller Myotomy And Gastric Bypass. Obesity surgery. 2010 Aug;20(8):1035-35.

9. Leon P, Csendes A, Braghetto I, Lasen D, Robles J. Achalasia in morbidly obese patients. Report of two cases. Revista Chilena De Cirugia. 2010 Apr;62(2):172-74.

10. O'Rourke RW, Jobe BA, Spight DH, Hunter JG. Simultaneous surgical management of achalasia and morbid obesity. Obesity surgery. 2007 Apr;17(4):547-9.

11. Kaufman JA, Pellegrini CA, Oelschlager BK. Laparoscopic Heller myotomy and Roux-en-Y gastric bypass: a novel operation for the obese patient with achalasia. Journal of laparoendoscopic & advanced surgical techniques Part A. 2005 Aug;15(4):391-5.

12. Almogy G, Anthone GJ, Crookes PF. Achalasia in the context of morbid obesity: a rare but important association. Obesity surgery. 2003 Dec;13(6):896-900.

13. Donatelli G, Cereatti F, Soprani A. Per Oral Endoscopic Myotomy for the Management of Achalasia in a Patient with Prior Lap Band, Sleeve Gastrectomy, and Roux-en-Y Gastric Bypass. Obesity surgery. 2021 Jun;31(6):2843-44.

14. Bomman S, Klair JS, Ashat M, El Abiad R, Gerke H, Keech J, et al. Outcomes of peroral endoscopic myotomy in patients with achalasia and prior bariatric surgery: A multicenter experience. Diseases of the esophagus : official journal of the International Society for Diseases of the Esophagus. 2021 Dec 24;34(12).

15. Kolb JM, Jonas D, Funari MP, Hammad H, Menard-Katcher P, Wagh MS. Efficacy and safety of peroral endoscopic myotomy after prior sleeve gastrectomy and gastric bypass surgery. World journal of gastrointestinal endoscopy. 2020 Dec 16;12(12):532-41.

16. Aiolfi A, Foschi D, Zappa MA, Dell'Era A, Bareggi E, Rausa E, et al. Laparoscopic Heller Myotomy and Dor Fundoplication for the Treatment of Esophageal Achalasia After Sleeve Gastrectomy-a Video Vignette. Obesity surgery. 2021 Mar;31(3):1392-94.

17. Kim D, Pullat R, Crowley N. Robotic Redo Heller Myotomy after Laparoscopic Heller Myotomy in a Patient with Recurrent Achalasia after a Roux-en-Y Gastric Bypass. The American surgeon. 2019 Mar 1;85(3):e162-e63.

18. Casas MA, Schlottmann F, Herbella FAM, Buxhoeveden R, Patti MG. Esophageal achalasia after Roux-en-Y gastric bypass for morbid obesity. Updates in surgery. 2019 Dec;71(4):631-35.

19. Aiolfi A, Tornese S, Barbieri L, Panizzo V, Micheletto G, Bona D. Laparoscopic Heller myotomy after Roux-en-Y gastric bypass. European Surgery-Acta Chirurgica Austriaca. 2019 Aug;51(4):220-23.

20. Sanaei O, Draganov P, Kunda R, Yang D, Khashab MA. Peroral endoscopic myotomy for the treatment of achalasia patients with Roux-en-Y gastric bypass anatomy. Endoscopy. 2019 Apr;51(4):342-45.

21. Birriel TJ, Claros L, Chaar ME. Laparoscopic Heller myotomy after previous Roux-en-Y gastric bypass. Surgery for obesity and related diseases : official journal of the American Society for Bariatric Surgery. 2017 Nov;13(11):1927-28.

22. Luo RB, Montalvo D, Horgan S. Peroral endoscopic myotomy after gastric bypass: An effective solution for de novo achalasia. Surgery for obesity and related diseases : official journal of the American Society for Bariatric Surgery. 2017 Feb;13(2):e1-e3.

23. Masrur M, Gonzalez-Ciccarelli LF, Giulianotti PC. Robotic Heller myotomy for achalasia after laparoscopic Roux-en-Y gastric bypass: a case report and literature review. Surgery for obesity and related diseases : official journal of the American Society for Bariatric Surgery. 2016 Nov;12(9):1755-57.

24. Nguyen D, Dip F, Lo Menzo E, Szomstein S, Rosenthal R. Heller oesophagomyotomy as treatment for achalasia after gastric bypass for morbid obesity. Annals of the Royal College of Surgeons of England. 2016 Jan;98(1):e3-5.

25. Johnson WD, Marshall MB. Surgical Management of Achalasia in a Patient With Previous Gastric Bypass. Innovations (Phila). 2016 May-Jun;11(3):214-6.

26. Boules M, Corcelles R, Zelisko A, Batayyah E, Froylich D, Rodriguez J, et al. Achalasia After Bariatric Surgery. Journal of laparoendoscopic & advanced surgical techniques Part A. 2016 Jun;26(6):428-32.

27. Torghabeh MH, Afaneh C, Saif T, Dakin GF. Achalasia 5 years following Roux-en-y gastric bypass. Journal of minimal access surgery. 2015 Jul-Sep;11(3):203-4.

28. Yang D, Draganov PV. Peroral endoscopic myotomy (POEM) for achalasia after Roux-en-Y gastric bypass. Endoscopy. 2014;46 Suppl 1 UCTN:E11-2.

29. Oh HB, Tang SW, Shabbir A. Laparoscopic Heller's cardiomyotomy and Roux-En-Y gastric bypass for missed achalasia diagnosed after laparoscopic sleeve gastrectomy. Surgery for obesity and related diseases : official journal of the American Society for Bariatric Surgery. 2014 Sep-Oct;10(5):1002-4.

30. Benavente-Chenhalls LA, Sherman V, Reardon PR. Laparoscopic Heller myotomy and gastric bypass for achalasia after vertical banded gastroplasty. Surgery for obesity and related diseases : official journal of the American Society for Bariatric Surgery. 2011 Sep-Oct;7(5):664-5.

31. Ramos AC, Murakami A, Lanzarini EG, Neto MG, Galvão M. Achalasia and laparoscopic gastric bypass. Surgery for obesity and related diseases : official journal of the American Society for Bariatric Surgery. 2009 Jan-Feb;5(1):132-4.

32. Cho M, Kaidar-Person O, Szomstein S, Rosenthal RJ. Achalasia after vertical banded gastroplasty for morbid obesity: A case report. Surgical laparoscopy, endoscopy & percutaneous techniques. 2006 Jun;16(3):161-4.

33. Murad MH, Sultan S, Haffar S, Bazerbachi F. Methodological quality and synthesis of case series and case reports. BMJ Evid Based Med. 2018 Apr;23(2):60-63.

34. Velotti N, Vitiello A, Berardi G, Musella M. Roux-en-Y Gastric Bypass and Heller Myotomy: One-Step Surgical Treatment of Symptomatic Achalasia in a Morbid Obese Patient. Obesity Surgery. 2021;31(7):3379-81.

35. Ithurralde-Argerich J, Rosner L, Faerberg A, Puma R, Ferro D, Cuenca-Abente F. Laparoscopic Heller Myotomy and Roux-en-Y Gastric Bypass as Treatment for Patients with Achalasia and Morbid Obesity: Outcomes in a Short Series of Patients. Journal of Laparoendoscopic & Advanced Surgical Techniques. 2021;31(1):29-35.

36. Friedman DT CA. Simultaneous laparoscopic Heller myotomy and roux-en-y gastric bypass in a morbidly obese patient with achalasia. Surg Endosc. 2017;31(1):S94.

37. Fisichella PM, Orthopoulos G, Holmstrom A, Patti MG. The surgical management of achalasia in the morbid obese patient. Journal of Gastrointestinal Surgery. 2015;19(6):1139-43.

38. Hagen ME, Sedrak M, Wagner OJ, Jacobsen G, Talamini M, Horgan S. Morbid obesity with achalasia: a surgical challenge. Obesity surgery. 2010;20(10):1456-58.

39. Pinto G PJ, Marin VD, Sendrea JG, Obregon F. Achalasia and morbid obesity: simultaneous management by Heller myotomy and gastric bypass. Obes Surg. 2010;20(8):1035.

40. Leon P CA, Braghetto I, Lasen D, Robles J. Achalasia in morbidly obese patients: report of two cases. Rev Chil De Cirugia. 2010;62(2):172-74.

41. O’Rourke RW, Jobe BA, Spight DH, Hunter JG. Simultaneous surgical management of achalasia and morbid obesity. Obesity surgery. 2007;17(4):547-49.

42. Kaufman JA, Pellegrini CA, Oelschlager BK. Laparoscopic Heller myotomy and Roux-en-Y gastric bypass: a novel operation for the obese patient with achalasia. Journal of Laparoendoscopic & Advanced Surgical Techniques. 2005;15(4):391-95.

43. Almogy G, Anthone GJ, Crookes PF. Achalasia in the context of morbid obesity: a rare but important association. Obesity surgery. 2003;13(6):896-900.

44. Donatelli G CFSA. Per Oral Endoscopic Myotomy for the Management of Achalasia in a Patient with Prior Lap Band, Sleeve Gastrectomy, and Roux-en-Y Gastric Bypass. Obesity Surgery. 2021;31:2843-44.

45. Bomman S KJ, Ashat M, El Abiad R, Gerke H, Keech J, Parekh K, Nau P, Hanada Y, Wong Kee Song LM, Kozarek R, Irani S, Low D, Ross A, Krishnamoorthi R. Outcomes of peroral endoscopic myotomy in patients with achalasia and prior bariatric surgery: A multicenter experience. Diseases of the Esophagus. 2021;34(12):doab044.

46. Aiolfi A, Tornese S, Bonitta G, Rausa E, Micheletto G, Bona D. Management of Esophageal Achalasia after Roux-en-Y Gastric Bypass: Narrative Review of the Literature. Obes Surg. 2019 May;29(5):1632-37.
